# Cognitive and motor cortex activation during robot-assisted multi-sensory interactive motor rehabilitation training: An fNIRS based pilot study

**DOI:** 10.3389/fnhum.2023.1089276

**Published:** 2023-02-09

**Authors:** Jinyu Zheng, Qiqi Ma, Wanying He, Yanping Huang, Ping Shi, Sujiao Li, Hongliu Yu

**Affiliations:** ^1^Institute of Rehabilitation Engineering and Technology, University of Shanghai for Science and Technology, Shanghai, China; ^2^Shanghai Engineering Research Center of Assistive Devices, Shanghai, China; ^3^Key Laboratory of Neural-Functional Information and Rehabilitation Engineering of the Ministry of Civil Affairs, Shanghai, China

**Keywords:** rehabilitation robot, multi-sensory interactive, cognitive cortex, motor cortex, near-infrared spectroscopy

## Abstract

**Objective:**

This study aimed to evaluate the effects of multiple virtual reality (VR) interaction modalities based on force-haptic feedback combined with visual or auditory feedback in different ways on cerebral cortical activation by functional near-infrared spectroscopy (fNIRS). Methods: A modular multi-sensory VR interaction system based on a planar upper-limb rehabilitation robot was developed. Twenty healthy participants completed active elbow flexion and extension training in four VR interaction patterns, including haptic (H), haptic + auditory (HA), haptic + visual (HV), and haptic + visual + auditory (HVA). Cortical activation changes in the sensorimotor cortex (SMC), premotor cortex (PMC), and prefrontal cortex (PFC) were measured.

**Results:**

Four interaction patterns all had significant activation effects on the motor and cognitive regions of the cerebral cortex (*p* < 0.05). Among them, in the HVA interaction mode, the cortical activation of each ROI was the strongest, followed by HV, HA, and H. The connectivity between channels of SMC and bilateral PFC, as well as the connectivity between channels in PMC, was the strongest under HVA and HV conditions. Besides, the two-way ANOVA of visual and auditory feedback showed that it was difficult for auditory feedback to have a strong impact on activation without visual feedback. In addition, under the condition of visual feedback, the effect of fusion auditory feedback on the activation degree was significantly higher than that of no auditory feedback.

**Conclusions:**

The interaction mode of visual, auditory, and haptic multi-sensory integration is conducive to stronger cortical activation and cognitive control. Besides, there is an interaction effect between visual and auditory feedback, thus improving the cortical activation level. This research enriches the research on activation and connectivity of cognitive and motor cortex in the process of modular multi-sensory interaction training of rehabilitation robots. These conclusions provide a theoretical basis for the optimal design of the interaction mode of the rehabilitation robot and the possible scheme of clinical VR rehabilitation.

## 1. Introduction

In the past two decades, research and development of robot-assisted rehabilitation have accelerated dramatically as a promising rehabilitation therapy (Cao et al., 2020). It provides a standardized environment for more intensive and repetitive interventions, thereby reducing the stress and workload of therapists. The basic design principle of rehabilitation robots is to induce cerebral cortex activation by processing external stimuli (Kaplan, 1988). If feedback stimuli related to motor performance are synchronized with motor output, these not only enhance motivation but also promote plasticity in the motor cortex (Stefan, 2000). External stimuli can be expressed in many forms, including visual, auditory, and haptic stimuli, in motor learning applications (Deutsch et al., 2004).

Virtual reality (VR) based rehabilitation therapy can provide various feedback stimuli such as visual, auditory, and haptic stimuli. Currently, its application in the clinical medical field is becoming increasingly widespread; however, its rehabilitation effects remain unclear. It has been argued that excessive feedback may lead to patient dependence. However, it has also been suggested that multisensory stimulation is beneficial for improving patients’ positive expectations and self-efficacy (Shamy, 2010). Therefore, to achieve the optimal training effect of robotic rehabilitation training, it is necessary to study the influence of the form and intensity of feedback stimuli on the method’s training effects. By exploring the optimal interaction mode, a theoretical basis for a robotic interaction design can be provided. This is of great significance for the development of cranial nerve rehabilitation.

Currently, most studies compare several single-feedback stimuli such as visual and auditory stimuli. Wang et al. (2022) studied the effect of visual and auditory feedback based on the upper limb rehabilitation system on cortical activation. However, haptic feedback was not involved in this study. Haptic feedback is the most direct and necessary form of motion information for robots (Lieberman and Breazeal, 2007), it is indispensable. Research methods that combine other types of feedback in a variety of ways based on haptic feedback appear to be more applicable and comprehensive. Therefore, this study explored the effects of haptic feedback combined with visual or auditory feedback on cortical activation. By exploring the optimal feedback method, the rehabilitation efficiency of a rehabilitation robot can be improved.

The basic principle of neurological rehabilitation for stroke is brain plasticity. In the process of rehabilitation training, external sensory stimulation can promote neural activity, thereby promoting neural remodeling and functional recovery. This is also the significance of VR technology used in stroke rehabilitation. Therefore, studying the neural activity of the brain during rehabilitation training is the most intuitive way to reflect the training effect. Typically, the motor and cognitive cortices are activated during robot-assisted rehabilitation training. The premotor cortex (PMC) is involved in the planning and execution of motor tasks (Grafton et al., 1998). The sensorimotor cortex (SMC) is associated with task complexity and attention (control of attentional resources) during voluntary movements (Han et al., 2018). The PFC is mainly responsible for executive control processes related to working memory, coordinating other brain regions to accomplish goal-oriented behaviors, and plays an essential role in higher cognitive functions such as episodic memory and reasoning ability (Jacky et al., 2015, Carlen, 2017). Studies have shown that neural networks are active during complex executive processes and that the PFC is highly correlated with the posterior parietal cortex (Periánez et al., 2004). Therefore, the PMC, SMC, and PFC were selected as regions of interest (ROIs) in this study. During the training process, the cortical activation of the ROIs was measured to investigate the impact of different interaction modes on the training effect.

Currently, functional neuroimaging techniques that can be used to explore the activation of the cortex by external stimuli include positron emission tomography (PET), functional magnetic resonance imaging (fMRI), and functional near-infrared spectroscopy (fNIRS). Among these, fNIRS is non-invasive, has moderate spatial resolution, allows participants to perform body movements, is easy to wear, and has low sensitivity to motion artifacts. It is suitable for experiments with strong demands for interaction or brain activity detection in natural situations. Therefore, this research used fNIRS technology to study the corresponding ROIs in the cerebral cortex. The rationale is that neuronal activity induces hemodynamic responses through neurovascular coupling, which are related to changes in oxyhemoglobin (HbO) and deoxyhemoglobin (HbR) concentrations measured by fNIRS (Scholkmann et al., 2014). Therefore, neuronal activity can be studied by observing changes in hemoglobin concentration in cerebral blood flow.

The purpose of this study was to use fNIRS technology to detect the activation of the cerebral cortex under different VR interaction modes and to explore the optimal feedback mode to improve the effectiveness of rehabilitation training. A multimodal, VR, interactive training system based on an end effector rehabilitation robot was developed to provide modular visual, auditory, and haptic feedback. In this study, experimental conditions based on haptic feedback that combined visual and auditory stimuli in different forms were established, including haptic (H), haptic + auditory (HA), haptic + visual (HV), and haptic + visual + auditory (HVA). This research method was used to compare and study the potential differential effects of different VR interaction modes on the cerebral cortex and to explore a better interaction method. This provided a theoretical basis for optimizing the interaction design of rehabilitation robots.

## 2. Materials and methods

### 2.1. Equipment

#### 2.1.1. VR interactive system of upper-limb rehabilitation robot

**FIGURE 1:**
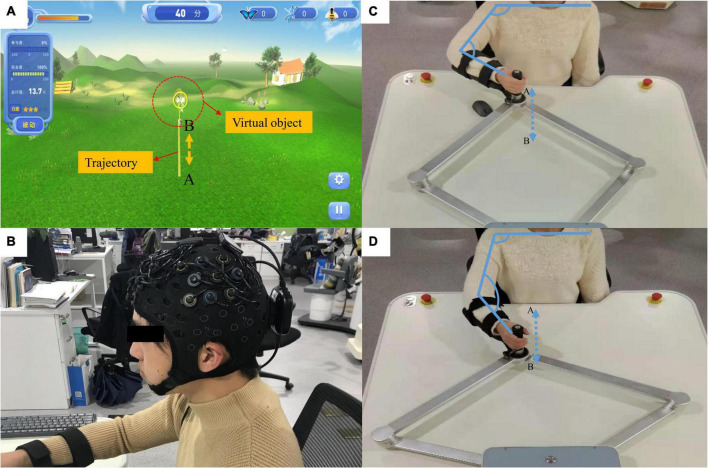
Rehabilitation robots and fNIRS equipment. **(A)** The virtual reality (VR) system; the yellow line (from A to B) represents the training trajectory while the butterfly net represents the virtual object mapping the robot’s end effector. **(B)** The fNIRS equipment. **(C,D)** The actual movement process during the experiment. The participant’s hand was fixed onto the end effector and they carried out a straight-line reciprocating motion from A to B. The actual distance from A to B is 0.45 meters.

The end effector upper-limb rehabilitation robot ArmGuider was jointly developed by the University of Shanghai for Science and Technology and Shanghai ZD Medical Technology Co., Ltd., Shanghai, China. It is mainly composed of a working platform, linkage mechanism, power system, and display screen (Figure 1). The working platform is 1.20 meters long and 1.10 meters wide. The screen is 0.94 meters long and 0.53 meters wide. The robot offers multiple training modes and different speed and intensity levels. Moreover, it can realize trajectory training in the horizontal plane. The two-link system can transmit the interactive force between the power system and the end effector as a transmission component. During the training process, the patient’s affected arm was secured to the end effector (Figures 1C, D). The patient applied a force or the force was driven by the end effector. The robot can adjust the strength of the assistance or resistance provided in real time according to the force exerted by the user on the end effector to maintain a constant speed of movement or the patient’s training motivation. The target disease for this rehabilitation robot is stroke with mild motor and cognitive impairment. Some previous studies have shown that for stroke patients with mild brain injury, the neural response of their cerebral cortex is similar to that of healthy people (Rehme et al., 2011, Rehme et al., 2012). Therefore, the cortical activity of healthy people can reflect the neural activity of patients with mild stroke to a certain extent, which provides a certain experimental basis for clinical treatment.

Several studies on robotic therapy devices have shown that continuous passive movement combined with active movement can promote motor recovery (Krebs et al., 2003, Hogan et al., 2006, Krebs, 2009). In this study, a novel passive-active combined training mode was adopted, as this mode allows for the adjustment of the interaction force according to the degree of patient participation to achieve the transformation of the active and passive modes. This innovative robotic training mode will assist therapists in delivering optimized therapy to restore upper extremity function in stroke patients with various needs and abilities (Blank et al., 2014). In addition, according to the characteristics of passive-active training, we designed a corresponding VR interaction system using the Unity 3D game engine. The virtual environment mainly uses natural scenery such as forests and grasslands as design elements (Figure 1A). The position of the end effector and the direction of the force were calculated in real-time and streamed to the virtual reality application. A butterfly net in a virtual environment then mapped the end effector. The butterfly flew according to a preset trajectory (Figure 1A) to represent the participants in the virtual environment and moved accordingly based on their actual movements.

In this study, auditory, visual, and haptic feedback were combined in a VR system. Haptic feedback involves the robot adjusting the force exerted on the end effector according to the active force exerted by the participant. To evaluate the patient’s exercise ability, we introduced the concept of “engagement,” that is, the proportion of the patient’s active exertion in the force required to complete the task. Throughout the training process, user engagement was displayed on the screen in real-time. The main task of the participants was to control the butterfly net to catch the virtual butterfly by pushing the end effector. When the participant’s engagement exceeded 30%, the butterfly in the virtual environment was “caught.” Visual feedback refers to the real-time display of the motion trajectory and special effects of bonus points when the task was completed. Auditory feedback refers to the sound played when a task was completed. Typically, feedback strategies can be categorized according to when feedback is provided: during motor task execution [concurrent (online, real-time) feedback], or after (terminal feedback) motor task execution. In general, a visual concurrent feedback design is desirable to guide participants to optimal movements without relying on feedback. The reference trajectories provided additional information regarding the participants’ range of motion. Therefore, concurrent feedback and terminal feedback were included in this study, and their effects on neural activity need to be further explored.

#### 2.1.2. fNIRS system

**FIGURE 2:**
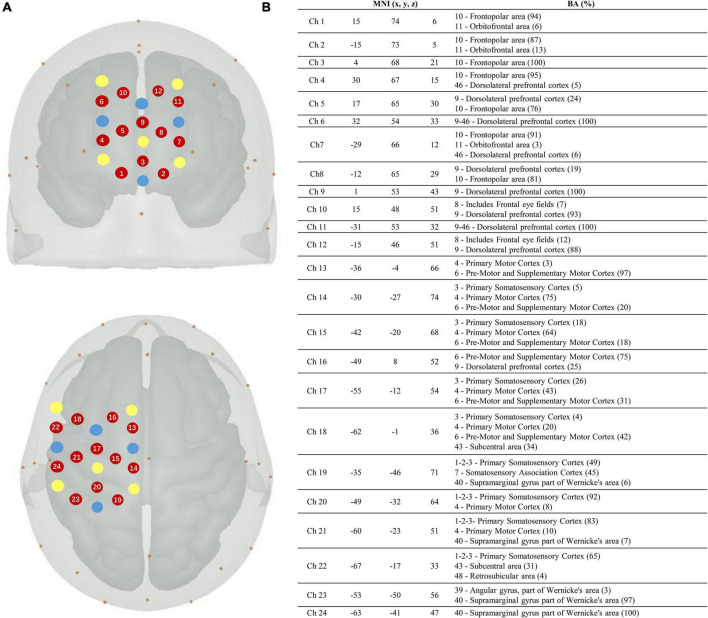
Localization of the fNIRS optodes, channels, MNI coordinates, and Brodmann correspondences. **(A)** Yellow, transmitters; blue, detectors; and red, channels. **(B)** Montreal Neurological Institute (MNI) coordinates for each channel (*n* = 24) with x, y, and z coordinates. The Brodmann area correspondences (number, name, and %) were extracted from the NIRS-SPM toolbox on the right.

In this study, a continuous-wave fNIRS system (Brite24, Artinis, Netherlands) was used to measure cortical activity. A system with wavelengths of 760 and 850 nm was used to record cortical activity at a sampling rate of 10 Hz. To test cortical neural activity in the cognitive and motor areas, we chose two 12-channel optode templates with a total of 18 optodes (10 light sources and 8 detectors) (Figure 2). The optodes were mounted on a holder on the NIRS cap. The distance between the sources and detectors was 3.0 cm. For accurate fixation, caps were available in large, medium, and small sizes to accommodate different head sizes. The international 10–20 system was used to locate the fNIRS optodes (Wang et al., 2018). Cz, Fz, and other symbol positions were marked on the caps according to the 10–20 system. For more accurate positioning, the cranial vertex (Cz) was set as a reference point for the positioning of the optodes. In addition, the Montreal Neurological Institute (MNI) 152 is the most widely used average brain template, created by averaging 152 brains co-registered with the Talairach brain (Peters, 1998) to eliminate differences in the shape and anatomy of different brains. Previous studies have established a correspondence between the MNI coordinate system and the 10–20 system (Jasper, 1958). At the same time, NIRS-SPM provides statistical parametric mapping tools for fNIRS (Collins et al., 1994). NIRS-SPM uses probabilistic registration of 3D spatial data of optodes and 10–20 landmark positions to transform functional images into the MNI space (Figure 2). The brain areas corresponding to each channel were then extrapolated from the reference points based on the MNI template (Okamoto et al., 2004). The ROIs based on the Brodmann area (BA) regions included the SMC (BA4), PMC (BA6), and PFC (BA8/9/46). The channels corresponding to each ROI were as follows: PFC: channels 1–12; SMC: channels 14, 15, 17, 19, 20, 21, and 23; and PMC: channels 13, 16, 18, 22, and 24.

### 2.2. Participants

Twenty healthy participants (five women; mean age, 24 ± 2.34) with no history of neurological, motor, or psychological disorders participated in this study. Auditory, visual, and cognitive abilities were tested during the experimental training before the experiment, and no impairments were found. All the participants were fully informed of the experimental procedures. In addition, to avoid the different effects on cortical activation that occur due to handedness, we tested the handedness of all participants using the Edinburgh Handedness Inventory to ensure the accuracy of the experimental results (Oldfield, 1969). The test results showed that all the participants were right-handed.

### 2.3. Procedure

**FIGURE 3:**
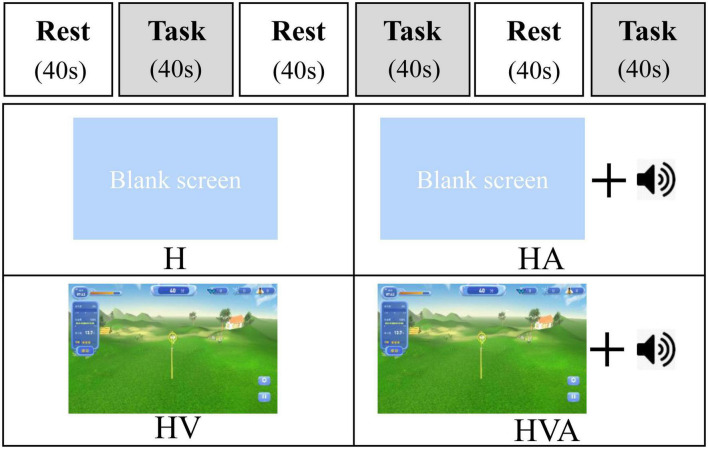
The experimental paradigm and the four different virtual reality (VR) training modes. The patients performed four cycling tasks, each starting with a 40 s rest period and a 40 s training period, which was repeated three times. The abbreviations in the figure represent haptic (H), haptic + auditory (HA), haptic + visual (HV), and haptic + visual + auditory (HVA).

In this study, the four experimental conditions were as follows: H, HA, HV, and HVA. As shown in Figure 3, under the H condition, the interactive interface was blank, and the participants could only feel the interactive force of the robot end effector without visual and auditory feedback. In the HA condition, interactive forces and prompt tones were provided, but no interactive interface was visible. Those in the HV and HVA conditions could see the interactive interface; however, the conditions differed in terms of whether a prompt tone was provided. In addition, the training speed was set to 0.12 m/s. The training trajectory was a straight line (length of 45 cm) in the Y-direction, as shown in Figures 1C, D. The trajectory allows for the training of elbow flexion and extension and strengthening of the biceps and triceps muscles. We measured the participants’ cortical activity under the four conditions. A block paradigm design that repeats three cycles (with each cycle consisting of two phases, rest (40 s) to task (40 s)) was used for each task, as depicted in Figure 3. Therefore, a single measurement lasted 240 s. During the 40 s task phase, there are approximately five upper arm flexion and extension movements. The execution sequence of the four experimental conditions was assigned randomly by applying a random permutation function “randperm” in MATLABR2012b (MathWorks, Natick, MA, USA).

Cortical activation was measured using an fNIRS system. The ROIs were the SMC, PMC, and PFC, and the arrangement of the optodes is illustrated in Figure 2. As the signals of channels 1–7 were weak and unstable after pre-processing, these channels were removed. The channels corresponding to each ROI were as follows: PFC, channels 8, 9, 10, 11, and 12; SMC, channels 14, 15, 17, 19, 20, 21, and 23; and PMC, channels 13, 16, 18, 22, and 24. According to the modified Beer–Lambert law, we obtained the HbO and HbR values that followed changes in cortical concentration levels (Holper et al., 2009). The optodes were placed on the cap according to the template. According to the international 10–20 system, the cap was positioned on each participant’s forehead by centering the specific mark on the bottom line of the optodes at the Fpz (10% of the distance between the Nasion and Inion). The same method was applied for Cz and Fz for validation. We then used the MNI template for probabilistic registration in the NIRS-SPM system (Collins et al., 1994). The luminous flux of each channel was adjusted to a better range to ensure the reliability of the experimental data.

The experiment was conducted in a quiet and stable-light environment. Before the experiment, the participants were asked to sit comfortably in a chair with their upper bodies upright. The heights of the working platform and chair were adjusted to a comfortable position. The participant’s right forearm was fixed to the end effector. Moreover, the participants were trained on the robot for five minutes to familiarize themselves with the experimental procedure and operating methods. During the experiment, the participants were required to perform upper limb reciprocation between points A and B under different experimental conditions (Figures 1C, D). Participants were required to actively participate to achieve the highest possible engagement. In addition, auditory stimuli were presented to participants through loudspeakers. The participants were required to focus on the screen in all four experimental conditions, including on the blank background in the H and HA conditions, as shown in Figure 3. In addition, participants were instructed to relax their bodies during the experiment and avoid physical movements other than those of the right arm, including facial movements, frequent blinking, and looking around. One experimenter operated the robot and provided participants with verbal prompts including “start” and “rest,” while another experimenter used the fNIRS system to monitor changes in cerebral cortex activity in real-time (Cope and Delpy, 1988).

### 2.4. Data analysis

#### 2.4.1. Cortical activation imaging

The fNIRS equipment measured changes in the optical density of cerebral blood flow non-invasively. According to the modified Beer–Lambert law, the relative change in HbO concentration can be obtained by changing the optical density (Holper et al., 2009). We analyzed the fNIRS data using the software package NIRS-SPM (KAIST, Daejeon, South Korea) (Collins et al., 1994) implemented in MATLAB (MathWorks, Natick, MA, USA). Global drifts often occur due to breathing, cardiac, vaso-motion, or other experimental errors in fNIRS experiments. In this study, the hemodynamic response function (HRF) and wavelet-MDL were employed to eliminate the global trend and improve the signal-to-noise ratio (SNR) (Yu et al., 2011). Besides, the hemodynamic modality separation method was used to further remove the global trend (Yamada et al., 2012). Moreover, a generalized linear model (GLM) was used to analyze the fNIRS data by simulating the hypothetical HbO response under experimental conditions (Ye et al., 2009). After analyzing each participant’s data, a group analysis was conducted on all participants’ experimental data under the same experimental conditions, and a *t*-test was selected to obtain the activation diagram of each experimental condition.

#### 2.4.2. Statistical analysis

The GLM model is a linear combination of predicted responses to different stimuli and error terms. By comparing the ideal and detected modes of the GLM, the β coefficient can be estimated by applying the least-squares method. The activation level of the cerebral cortex can then be obtained by statistical analysis of the β (Collins et al., 1994). Each channel corresponds to a β value that represents the activation level of the channel. The β value of the corresponding channel in each ROI was statistically analyzed as a parameter representing the activation level of this channel. In this study, the average of the β values of channels located in the same ROI was calculated and then a two-way repeated measures ANOVA was performed across different experimental conditions and different ROIs. In addition, a Greenhouse–Geisser (G–G) correction was applied when the spherical hypothesis was violated. The Bonferroni test was used for *post hoc* analysis, followed by ANOVA. Statistical analysis was performed using SPSS software for Windows (version 26.0; SPSS Inc., Chicago, IL, USA). If the *p*-value was less than 0.05, the null hypothesis of no difference was rejected.

#### 2.4.3. Connectivity analysis

Connectivity analysis provides more information regarding dynamic network-level changes than that inferred from the extent and laterality of activation (Veldsman et al., 2015). This may be a complementary approach to understanding the neural reorganization patterns underlying stroke recovery (Grefkes and Fink, 2014). In this study, the Pearson correlation coefficient was used to characterize the connectivity among the studied brain areas. The Pearson correlation coefficient was obtained by dividing the covariance by the standard deviation of the two variables (generally represented by *r*), with *r* represented by values between −1 and 1. As the linear relationship between the two variables increased, the correlation coefficient tended to be 1 or −1. The HbO concentration in the task phase was analyzed, and the sample size for each channel was 1200. Pearson correlation coefficients between each channel were calculated. The average of the analysis data for all participants was then computed. In order to compare the correlation coefficients more intuitively, statistical analysis was carried out. The average value of correlation coefficients of between channels located in the corresponding ROIs was calculated. Then, one-way ANOVA was performed among the experimental conditions, and the Bonferroni correction was conducted.

## 3. Results

This study investigated the cortical activation and functional connectivity among brain areas during different VR interaction modes using fNIRS technology. The engagement was calculated as the behavioral result. Optical imaging and statistical analysis of beta waves were performed for cortical activation analysis. In addition, Pearson’s correlation analysis was applied to assess the connectivity among the ROIs.

**FIGURE 4:**
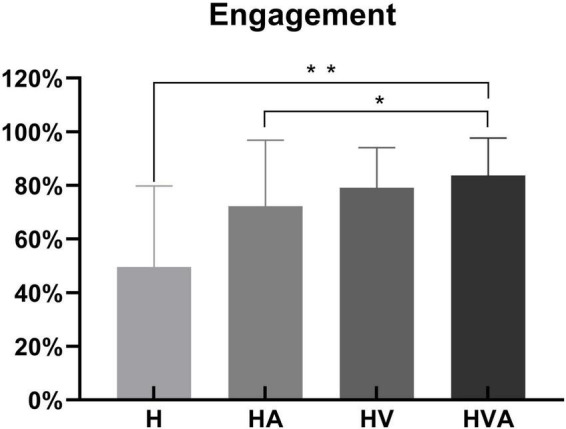
Statistical analysis of the engagement under four experimental conditions. *p < 0.05, **p < 0.01. The abbreviations in the figure represent haptic (H), haptic + auditory (HA), haptic + visual (HV), and haptic + visual + auditory (HVA).

The statistical analysis of engagement in four conditions is shown in Figure 4. During the experiment, the sensors of the robot recorded the active force exerted by the subject on the end effector. We calculated the ratio of the subject’s active force to the total force required to complete the task to obtain the engagement, as the behavioral result. One-way ANOVAs were performed for the engagement of four conditions. As can be seen in Figure 4, the engagement was highest in the HVA group, followed by the HV, HA, and H groups, respectively. Among them, there were significant differences between the H and HVA, HA and HVA conditions (*p* < 0.05).

**FIGURE 5:**
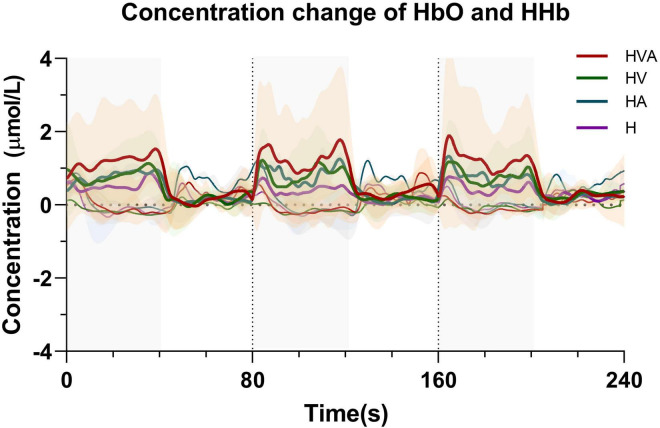
Time series of oxyhemoglobin (HbO) and HHb changes during the task. The solid line represents the mean of the concentration and the shade represents the error (mean ± SD) (*n* = 20). The three areas separated by dotted lines in the figure represent three trials, where the first 40 s of each trial are the task phase. The red, green, blue, and purple curves represent HVA, HV, HA, and H modes, respectively. Thicker lines represent HbO and thinner lines represent HHb. The abbreviations in the figure represent haptic (H), haptic + auditory (HA), haptic + visual (HV), and haptic + visual + auditory (HVA).

The time series data of concentration change of HbO and HHb are plotted in Figure 5. We randomly selected channel 8 and calculated the averaged fNIRS responses (of both HbO and HHb) that were superimposed across four conditions of all subjects. The solid line represents the mean of the concentration and the shade represents the error (mean ± SD) (n = 20). The three areas separated by dotted lines in the figure represent three trials, where the first 40 s of each trial are the task phase. In the figure, HbO and HHb under the same conditions are drawn in the same color. In the task phase, the HbO concentration change curve shows an upward trend, while the HHb concentration change curve shows a downward trend. It can be seen from the figure that the average concentration changes of HbO and HHb show good periodicity. When the concentration of HbO increases, the concentration of HHb decreases slightly.

**FIGURE 6:**
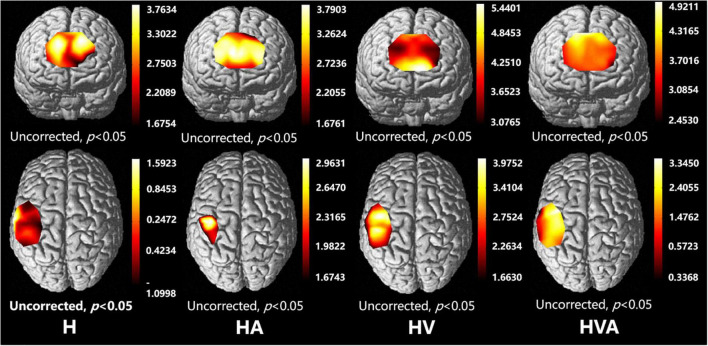
Optical imaging of cortical activities (group analysis). The abbreviations in the figure represent haptic (H), haptic + auditory (HA), haptic + visual (HV), and haptic + visual + auditory (HVA).

The fNIRS cortical activation imaging scans during the four VR training modes are illustrated in Figure 6 (*p <* 0.05, uncorrected). The color bar represents the *t*-value. As shown in the figure, the SMC, PMC, and PFC regions showed significant activation (*p <* 0.05) under the four interaction modes. It is worth mentioning that the activation levels in both the cognitive and motor regions of the HV and HVA groups were similar. Furthermore, compared to the H and HA groups, the activation area, and degree of the HVA and HV groups were stronger. Additionally, in the motor region, the activation area of the H group was broader than that of the HA group. Nevertheless, the degree of activation of the HA group was higher than that of the H group (Figure 6).

**FIGURE 7:**
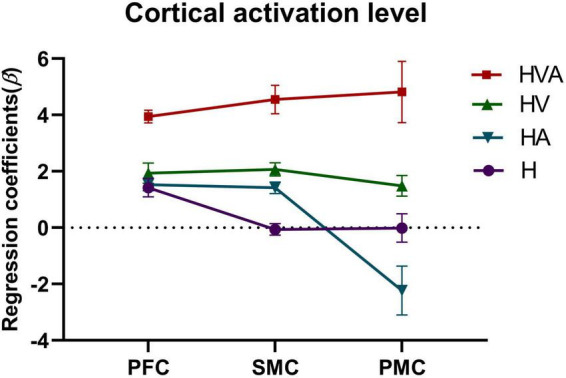
Statistical analysis of the regression coefficients (β) under four experimental conditions of three regions of interest (ROIs). SMC, sensorimotor cortex; PFC, prefrontal cortex; PMC, premotor cortex. The abbreviations in the figure represent haptic (H), haptic + auditory (HA), haptic + visual (HV), and haptic + visual + auditory (HVA).

The statistical analysis of the regression coefficients (β) under four experimental conditions of three ROIs was shown in Figure 7. The results of repeated measures ANOVA showed that the main effect of experimental conditions was significant, *F* = 27.4, *p* < 0.001, η^2^ = 0.259. The main effect of ROI was significant, *F* = 5.705, *p* = 0.012, η^2^ = 0.024. The interaction between ROI and conditions was significant, *F* = 4.686, *p* = 0.001, η^2^ = 0.056. The simple effect test of experimental conditions showed that in PFC, the simple effect of experimental conditions was significant, *F* = 26.216, *p* < 0.001, η^2^ = 0.251. In SMC, the simple effect of experimental conditions was significant, *F* = 27.701, *p* < 0.001, η^2^ = 0.261. In PMC, the simple effect of experimental conditions was significant, *F* = 11.156, *p* < 0.001, η^2^ = 0.125. The simple effect test result of ROI shows that under the H condition, the simple effect of ROI was significant, *F* = 8.440, *p* < 0.001, η^2^ = 0.067. Under HA condition, the simple effect of ROI was significant, *F* = 9.155, *p* < 0.001, η^2^ = 0.073. Under the HV condition, the simple effect of ROI was not significant, *F* = 0.243, *p* = 0.785, η^2^ = 0.002. Under the HVA condition, the simple effect of ROI was not significant, *F* = 1.604, *p* = 0.203, η^2^ = 0.014. After multiple comparisons, it was found that under the H condition, the beta values of PFC, PMC, and SMC decreased in turn, and the beta values of PFC were significantly higher than SMC (*p* < 0.001). Under the HA condition, the beta values of PFC, SMC, and PMC decreased in turn, and the beta values of PFC and SMC were significantly higher than that of PMC (*p* < 0.001). Under the HV condition, the beta values of SMC, PFC, and PMC decreased in turn, without significant difference. Under the HVA condition, the beta values of PMC, SMC, and PFC decreased in turn, and there was no significant difference.

**FIGURE 8:**
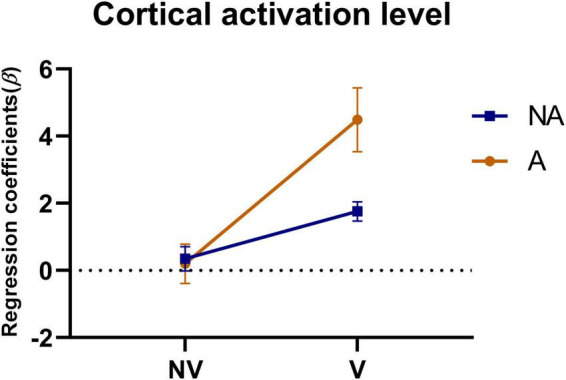
Statistical analysis of the regression coefficients (β) under the presence or absence of visual feedback or auditory feedback. A simple representation was given in the figure. NV, no visual feedback; V, visual feedback; NA, no auditory feedback; A, auditory feedback.

To investigate the effect of auditory feedback in the presence of visual feedback, we conducted a two-way ANOVA on the beta values of all ROIs under the condition of the presence or absence of visual and auditory feedback. The results of the intersubjective effect test showed that the test statistic of whether there was visual feedback or not was *F* = 86.472, *p* < 0.001, indicating that there was a significant difference in the effect of visual feedback on the activation level. The test statistic of auditory feedback was *F* = 17.633, *p* < 0.001, indicating that there was a significant difference in the influence of auditory feedback on the activation level. The test statistic of whether there was visual feedback * whether there was auditory feedback was *F* = 22.168, *p* < 0.001, indicating that visual and auditory feedback have an interaction effect, which had a significant impact on the activation level. The result of the descriptive statistical analysis was shown in Figure 8. As can be seen from the figure, in the absence of visual feedback, the impact of auditory feedback on the activation level was similar, while in the presence of visual feedback, the impact of auditory feedback on the activation level was significantly improved.

The functional connectivity analysis of HbO among the ROIs is shown in Figure 6. Each pixel value in the 24 × 24 matrix corresponds to the value of the Pearson correlation coefficient, which represents the correlation between the two measurement channels. The channels were ordered according to the ROI to which they belong. The ROIs were distinguished by gaps forming a 9 × 9 matrix. The numbers marked in the figure are the mean values of the Pearson correlation coefficients among the channels in each ROI. It can be concluded that the correlation between the SMC and PMC was the strongest in the HVA training mode, whereas the strongest correlation between the PMC and PFC was found in the H training mode. The strongest correlation between the SMC and PFC was observed in the HVA and HV training modes. In addition, the correlations between the channels within the PFC and SMC were stronger in the H and HA modes. However, the correlation between the channels of the two ROIs was stronger under the HV and HVA modes.

The results of the statistical analysis of the correlation coefficients (r) are shown in Figure 10. As can be seen from the figure, the connectivity of bilateral PFC vs. PFC, and SMC vs. SMC was strong and similar under all experimental conditions. The connectivity of bilateral PFC vs. SMC, and PMC vs. PMC was stronger under HVA and HV conditions, and the connectivity of SMC vs. PMC was strongest under HVA conditions. However, the connectivity of bilateral PFC vs. PMC was the strongest under H.

## 4. Discussion

**FIGURE 9:**
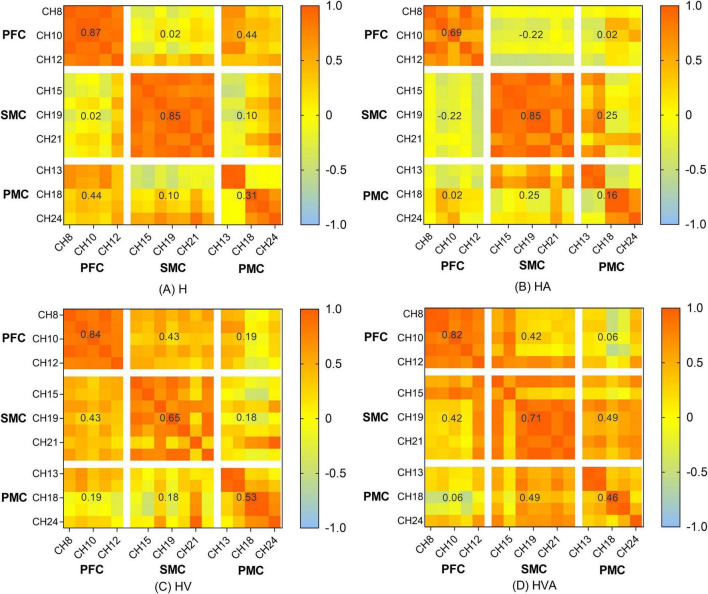
Heat map of the connectivity among the channels. The matrix map includes all channel pairs. Color bars indicate the value of *r*. The x and y axes representing the ROIs indicate the dorsolateral prefrontal cortex (PFC, channels 8–12), somatosensory area (SMC, channels 14, 15, 17, 19–21, and 23), and premotor cortex (PMC, channels 13, 16, 18, 22, and 24). The abbreviations in the figure represent haptic (H), haptic + auditory (HA), haptic + visual (HV), and haptic + visual + auditory (HVA).

**FIGURE 10:**
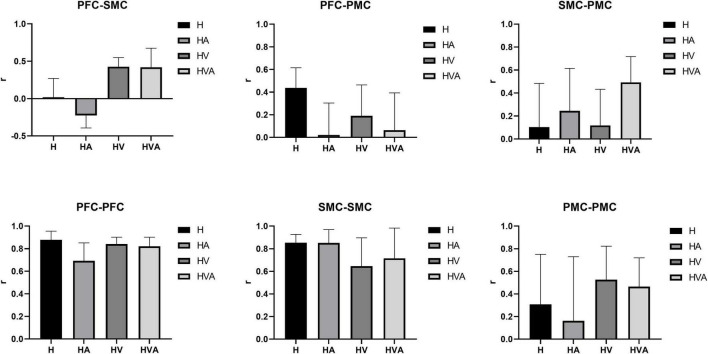
Statistical analysis of the correlation coefficients (r) under four experimental conditions between different regions of interest (ROIs). The ROIs include the bilateral dorsolateral prefrontal cortex (PFC, channels 8–12), somatosensory area (SMC, channels 14, 15, 17, 19–21, and 23), and premotor cortex (PMC, channels 13, 16, 18, 22, and 24). The abbreviations in the figure represent haptic (H), haptic + auditory (HA), haptic + visual (HV), and haptic + visual + auditory (HVA).

This study aimed to investigate the effects of different VR interaction modes on the degree of cortical activation and connectivity among ROIs. We developed a modular VR interactive system based on an end-effector rehabilitative robot. Four different VR interaction modes (H, HA, HV, and HVA) were included in this study, the behavioral performance and cortical activation were measured using the robot and fNIRS equipment, respectively. The results showed that the behavioral performance under HVA was the best (Figure 4). The average concentration changes of HbO and HHb showed good periodicity. When the concentration of HbO increases, the concentration of HHb decreases slightly (Figure 5). The results showed that all four VR interaction modes had significant activation effects on the cerebral cortex, with the HVA condition inducing the strongest activation, encompassing the SMC, PMC, and PFC regions (Figure 7). Furthermore, the HVA mode also displayed a significant advantage in functional connectivity between SMC and PMC regions (Figures 9, 10).

Previous research has shown that the visual perception of spatial information is more accurate, whereas the auditory perception of time information is even more accurate (Freides, 1974). Haptic perception can fulfill the relatively high requirements related to temporal and spatial information processing (Nesbitt, 2004), while visual feedback plays an essential role in therapeutic regimens for voluntary movements (Luara et al., 2016). These features can be powerful when employed with VR technology (Maciejasz et al., 2014). In addition, auditory feedback can redistribute one’s perceptual and cognitive load and become the focus of attention (Eldridge, 2006, Secoli et al., 2011). However, the impact of auditory feedback is largely dependent on the intuitiveness and accuracy of the mapping interpretation, and it must be chosen carefully; therefore, auditory displays are less common than visual displays (Sigrist et al., 2013). It can be seen from the results of this study that in the absence of visual feedback, compared with the H mode, the HA mode had a smaller area of activation in the motor cortex (Figure 6), a weaker degree of cortical activation in PMC (Figure 7), and weaker connectivity between PFC and SMC, PFC and PMC (Figures 9, 10). The results of two-way ANOVA of the existence of visual feedback and auditory feedback show that auditory feedback was difficult to show a strong effect on cortical activation level in the absence of visual feedback. However, in the presence of visual feedback, the improvement of activation level by fusion auditory feedback was significantly higher than that without auditory feedback, which may be related to the interaction effect between visual and auditory feedback (Figure 8).

As shown in Figures 6, 7, the HV and HVA groups demonstrated higher degrees of activation in the SMC and PFC regions than the H and HA groups. Previous studies have shown that the PFC is associated with decision-making and motor strategy development. Specifically, the PFC assists in regulating the response and behavior generated by environmental stimuli (Wood and Grafman, 2003) and participates in the attentional demands of trajectory planning. Thus, activation of the PFC reflects its role in maintaining attention and regulating postural control (Woollacott and Shumway-Cook, 2002, Drew et al., 2004). In contrast, the SMC plays a vital role in the early stages of motor learning and is mainly involved in observing motor tasks and integrating multiple sensory inputs (Bhattacharjee et al., 2020). It is usually activated in response to somatosensory stimuli such as haptic stimuli, disturbances, and passive movements. Thus, activation of the SMC and PFC regions during active upper-limb training represents the increased attention of the participants. As can be seen from the results of this study, interaction patterns that incorporate visual feedback may help to engage the attention of users.

As shown in Figure 7, the cortical activation levels were similar under the HV and HA interaction modes in SMC and PFC regions, whereas activation under the HVA condition was significantly higher than in the other interaction modes. This indicates that the higher activation levels under HVA conditions may not result from individual visual or auditory feedback; instead, these may result from a combination of multiple feedback modalities. Similar results have been reported in previous studies. For example, Leff et al. (2011) showed that the motion control system of the arm can adapt to a kinematic environment using auditory feedback and that the effect of auditory feedback is similar to that of visual feedback. Furthermore, Radziun and Ehrsson (2018) hypothesized that neuron populations integrate auditory signals with visual, tactile, and proprioceptive signals from the upper limbs, suggesting that the four interactions among vision, touch, proprioception, and sound are more conducive to the perception of limb ownership. In addition, studies have shown that auditory stimuli are effective at perceiving speed, regularity, and periodicity of motion (Kapur et al., 2005). Therefore, in future designs, auditory feedback design objects can incorporate the carrier signal, loudness, and pitch height (Konttinen et al., 2010).

As shown in Figures 9, 10, the correlation between the SMC and PMC was the strongest under the HVA mode. Notably, the PMC is responsible for motor initiation and motor control coding of skilled motor sequences (Sabes, 2000, Inoue and Sakaguchi, 2014). This suggests that HVA training modalities can provide sufficient feedback to stimulate the motor cortex to better facilitate motor initiation and control, which may be advantageous in the early stages of motor activity. In addition, some studies have shown that multimodal stimuli are generally perceived more accurately and faster than unimodal stimuli, reaching the threshold of neural activation earlier (Forster et al., 2002, Shams and Seitz, 2008). As task complexity increases, users prefer multimodal interactions, suggesting that users self-manage by shifting from unimodal interactions to multimodal interactions as their cognitive demands increase (Oviatt et al., 2004). Previous studies have shown that when visual information is input, the movement pattern is controlled according to the target location. In response, the movement policy responds quickly. In the absence of visual input, the response is slower but easier to recall later (Kovacs et al., 2010). Therefore, multimodal fusion should be employed during the early stages of motor learning. As the patient’s motor function continues to improve, the stimulation can be gradually reduced to maintain a greater cognitive load and enhance memory.

In summary, we studied the cortical activation patterns under four different VR training modes based on end-effector upper-limb rehabilitation robots. The results show that upper-limb rehabilitation robot training can activate the lateral SMC, PMC, and PFC. In addition, the HVA training mode displayed higher levels of brain activation and stronger connectivity among cortical regions. These results may contribute to the development of rehabilitation robots and provide a physiological basis for robot design and rehabilitation strategy formulation. Moreover, fNIRS can be a useful tool for studying the cortical effects of rehabilitation robots.

The limitations of this study must be considered. First, the form of auditory feedback used in this study was relatively simple and may not have strongly excited the nerves, thus affecting the experimental results. Second, some researchers have suggested that after training with multimodal stimuli, multimodal processing may be activated even if only a single modal stimulus is present (Shams and Seitz, 2008), which may have influenced the results of the experiments. In addition, the target users of this technology are older adults and people with upper extremity dysfunction; however, this hypothesis was tested only in young, healthy participants, and the number of participants was small. In the future, more participants should be included, including patients with neurological injuries, to investigate these initial findings in greater depth.

In the future, we will increase the modalities of robotic auditory stimulation to explore whether multiple auditory feedback tones lead to higher neural activity levels. In addition, elderly people and people with brain injuries will be included. Furthermore, the effects of other training modes, forces, and trajectories of the rehabilitation robot on cortical activation should be investigated to provide more comprehensive and systematic evidence.

## 5. Conclusion

In this study, we used fNIRS to investigate the significant activation of the parietal and prefrontal cortices during a VR training task. We have integrated visual and auditory feedback based on haptic feedback to form a multilevel VR training mode. With the integration of more sensory feedback, neuronal activity generally increased, which was reflected in the degree of cortical activation and connectivity of the ROIs. This indicates that multimode VR is more helpful in activating the cerebral cortex and promoting the connection of brain regions. The results may provide a specific theoretical basis for the human-computer interaction design of upper limb rehabilitation robots and provide an optimal interactive mode for rehabilitation robots.

## Data availability statement

The raw data supporting the conclusions of this article will be made available by the authors, without undue reservation.

## Ethics statement

The studies involving human participants were reviewed and approved by University of Shanghai for Science and Technology. The patients/participants provided their written informed consent to participate in this study.

## Author contributions

JZ and PS designed the experiment. WH and QM collected the data. JZ and YH analyzed the data and prepared all figures. JZ drafted the manuscript. SL and HY provided critical revisions. All authors approved the manuscript to be published.
